# Targeting autonomic flexibility to enhance cognitive training outcomes in older adults with mild cognitive impairment: study protocol for a randomized controlled trial

**DOI:** 10.1186/s13063-021-05530-z

**Published:** 2021-08-23

**Authors:** Feng V. Lin, Kathi Heffner, Richard Gevirtz, Zhengwu Zhang, Duje Tadin, Anton Porsteinsson

**Affiliations:** 1 Department of Psychiatry and Behavioral Sciences, School of Medicine, Stanford University, Stanford, USA; 2 Wu Tsai Neuroscience Institute, Stanford University, Stanford, USA; 3 Elaine C. Hubbard Center for Nursing Research on Aging, School of Nursing, University of Rochester Medical Center, Rochester, USA; 4 Department of Brain and Cognitive Sciences, University of Rochester, Rochester, USA; 5 Department of Medicine, School of Medicine and Dentistry, University of Rochester Medical Center, Rochester, USA; 6 Department of Psychiatry, School of Medicine and Dentistry, University of Rochester Medical Center, Rochester, USA; 7 Alliant International University, San Diego, USA; 8 University of North Carolina-Chapel Hill, Chapel Hill, USA; 9 Department of Neuroscience, School of Medicine and Dentistry, University of Rochester Medical Center, Rochester, USA

**Keywords:** Autonomic nervous system, Central autonomic network, Mild cognitive impairment, Biofeedback training

## Abstract

**Importance:**

Cognitive training with components that can further enhance the transferred and long-term effects and slow the progress of dementia is needed for preventing dementia.

**Objective:**

The goal of the study is to test whether improving autonomic nervous system (ANS) flexibility via a resonance frequency breathing (RFB) training will strengthen the effects of a visual speed of processing (VSOP) cognitive training on cognitive and brain function, and slow the progress of dementia in older adults with mild cognitive impairment (MCI).

**Design:**

Stage II double-blinded randomized controlled trial. The study was prospectively registered at ClinicalTrials.gov, with registration approved on 21 August 2020 (No. NCT04522791).

**Setting:**

Study-related appointments will be conducted on-site at University of Rochester Medical Center locations. Data collection will be conducted from August 2020 to February 2025.

**Participants:**

Older adults with MCI (*n* = 114) will be randomly assigned to an 8-week combined intervention (RFB+VSOP), VSOP with guided imagery relaxation (IR) control, and a IR-only control, with periodical booster training sessions at follow-ups. Mechanistic and distal outcomes include ANS flexibility, measured by heart rate variability, and multiple markers of dementia progress. Data will be collected across a 14-month period.

**Discussion:**

This will be among the first RCTs to examine in older persons with MCI a novel, combined intervention targeting ANS flexibility, an important contributor to overall environmental adaptation, with an ultimate goal for slowing neurodegeneration.

**Trial registration:**

ClinicalTrials.gov NCT04522791. Registered on 21 August 2020

Protocol version: STUDY00004727; IRB protocol version 2, approved on 30 July 2020.

## Introduction

Cognitive interventions are one of two primary categories of non-pharmacological interventions that have potential for slowing the progress of dementia [1]. So far, the transferred and long-term benefits of cognitive training in groups at risk for dementia [e.g., mild cognitive impairment (MCI)], however, have been limited. Cognitive intervention theories emphasize the importance of a prolonged “mismatch” between organismic supply (e.g., a person’s brain capacity) and environmental demands (e.g., cognitive challenges) for inducing neuroplasticity. To reach an ideal “mismatch,” the complexity of an intervention needs to increase in response to performance improvement in order to constantly exceed brain capacity [2].

Older adults with MCI face ongoing challenges related to their cognitively demanding everyday activities (e.g., managing finances, remembering names, etc.) [3]. Disrupted capacity to flexibly adapt to these challenges can exacerbate poor cognitive and brain health [4]. Adaptation capacity—that is, the ability to respond flexibly to environmental demands or stressors—is a key contributor to the neuroplasticity underlying broad and sustained effects of cognitive interventions [2]. Adaptation capacity is supported by central autonomic networks [(CAN), e.g., salience network (SN), somatosensory network (SAN)], which regulate homeostatic processes including the hypothalamic-pituitary-adrenal (HPA) axis, peripheral output of the autonomic nervous system (ANS) and other metabolic systems, and behavioral responses [5]. ANS flexibility reflects the integrity of central and peripheral systems that support adaptation to environmental demands, thereby serving as a key indicator of and contributor to adaptation capacity [6]. A decline in ANS flexibility is associated with a broad range of cognitive deficits and neurodegeneration, while enhancing ANS flexibility can widely and substantially strengthen cognitive and brain function [7–10]. We suggest that adaptation capacity, by supporting responsive and adaptive changes to environmental demands, undergirds neuroplasticity. Therefore, adaptation capacity is critical for promoting neuroplasticity underlying various cognitive enhancement activities (e.g., cognitive training) [2]. Our recently completed study found that ANS flexibility at baseline predicted the training effect of a cognitive training [11]. Hence, enhancing adaptation capacity may enhance neuroplasticity and slow the progress of dementia in older adults with MCI.

There are several categories of interventions that can explicitly improve ANS flexibility, including physical exercise [12, 13], non-invasive brain stimulation [14–16], and biofeedback intervention [17]. Mechanisms underlying the improvement include modifying the central efficiency of ANS flexibility, stimulating activity of the baroreflex, or regulating dopaminergic and cholinergic transmission [15, 18]. The effect sizes of these interventions on central and peripheral pathways of ANS flexibility vary: so far, the most robust intervention effect is from HRV biofeedback intervention (HRVB). HRVB entails feeding back beat by beat heart rate data during slow breathing maneuvers such that the participant tries to maximize peripheral nervous system function (indexed by high-frequency heart rate variability (HF-HRV)). The key behavioral component of HRVB is resonance frequency (RFB), that is, breathing at a rate which maximizes respiratory sinus arrhythmia (RSA), the synchronization of HR change (increases/decreases) with respiratory (inhalation/exhalation) patterns, creating a sine-wave-like curve of peaks and valleys. Multiple mechanisms are involved in explaining the effects of RFB on ANS, including (1) phase relationships between HR oscillations and breathing at specific frequencies (i.e., maximizing RSA), (2) phase relationships between heart rate and blood pressure oscillations at specific frequencies, (3) activity of the baroreflex, and (4) resonance characteristics of the cardiovascular system, all of which have been previously reviewed comprehensively [19]. Individuals can be easily trained to engage in RFB via an assistive mobile app that guides the individual, with a visual pacer, through a paced breathing exercise. A recent meta-analysis revealed an effect size of Hedges’ *g* = 0.83 for comparing HRVB (ranges 1–50 sessions) to other control interventions on improving adaption to real-world stressors (e.g., perceived stress and anxiety trait and state) [17]. Although RFB has not been tested in groups at risk for dementia, HRVB using RFB training improves resting HRV, executive function, and anxiety in traumatic brain injury [20, 21] and older adults [22]. HRVB also improves HRV response to cognitive stress [23]. We propose that adding RFB to a cognitive training can strengthen the effect on ANS flexibility and overall adaptation capacity, which in turn may directly modify markers of dementia progression, and may also indirectly enhance individuals’ adaptation to cognitive challenges provided by the cognitive training and strengthen cognitive training effect on markers of dementia. Lastly, compared to other intervention strategies for modifying ANS flexibility (e.g., vagal nerve stimulation, exercise, non-invasive brain stimulation), we suspect our proposed intervention approach, due to the minimal side effects, self-administrable nature, and potential effectiveness, may ensure a fast translation into real-world practice.

The objective of the study is to test whether adding RFB to the vision-based speed of processing training (VSOP)—the most widely tested cognitive training among older adults [24–27]—will enhance the training effects of VSOP by strengthening adaptation capacity that affords greater adaptive learning and neuroplasticity, and slow the progress of dementia in MCI. We will examine long-term effects of the combined intervention on ANS flexibility and cognitive and functional capacity and explore potential long-term effects of the combined intervention on neurodegeneration. Relevant hypotheses include the following: the combined intervention will have a greater effect on CAN and HRV—indicators of autonomic flexibility—as well as cognition and everyday function, and less neurodegeneration, compared to control groups throughout the follow-up period; improvements in ANS flexibility will predict improvement on cognition and everyday function and slower neurodegeneration over time.

## Methods

### Trial design

We plan to conduct a double-blinded, phase II randomized controlled trial (RCT). Older adults with MCI (*n* = 114) will be randomly assigned to an 8-week combined intervention (RFB+VSOP), VSOP with imagery-guided relaxation control (IR+VSOP), or a IR-only control, with 2-week booster sessions provided at 3 and 9 months after the intervention. Variables include ANS flexibility measured by central autonomic networks (central pathway) and heart rate variability (HRV; peripheral pathway) at rest and in response to a cognitively challenging task, and multiple markers of dementia progress, including cognition (battery tests of episodic memory and executive function), neuropsychiatric symptoms (NPS), instrumental activities of daily living function (IADL), and neurodegeneration (blood-based Alzheimer’s pathological markers and T1 cortical thickness measure). Data will be collected across a 14-month period: baseline and up to 3 time points after the intervention (immediately, 6 months, and 12 months after the intervention). An overview of the trial design is provided in Table 1.

**Table 1:** Overview of the study design

	Enrollment	Allocation	Post-allocation																		Close-out
Timepoint	***-1m***	0	***1m***	***2m***	***3m***	***4m***	***5m***	***6m***	***7m***	***8m***	***9m***	***10m***	***11m***	***12m***	***13m***	***14m***	***15m***	***16m***	***17m***	***18m***	***19m***
**Enrolment**	X																				
**Eligibility screen**	X																				
**Informed consent**	X																				
**Allocation**		X																			
**Interventions**																					
**RFB+VSOP**			X	X	X	X	X	X				X						X			
**IR+VSOP**			X	X	X	X	X	X				X						X			
**IR only**			X	X	X	X	X	X				X						X			
**Assessments**																					
**ANS flexibility**		X							X						X						X
**Cognition**		X							X						X						X
**NPS**		X							X						X						X
**IADL**		X							X						X						X
**Neurodegeneration markers**		X							X						X^a^						X

Note. ^a^Blood will not be collected during this point

### Trial status

The trial presented here is aligned with IRB protocol version 2, approved on 30 July 2020. The recruitment started on 18 August 2020 and is anticipated to be completed on 30 June 2024.

### Study setting

Study-related appointments will be conducted on-site at University of Rochester Medical Center locations. Subjects will self-administer interventions daily at home; in addition, there will be a weekly in-lab intervention session. All in-person assessments, including MRI appointments, for this research study will be conducted at the Rochester Center for Brain Imaging/Center for Advanced Brain Imaging and Neurophysiology (CABIN). Blood collection will be conducted at URMC’s Clinical Research Center (CRC).

### Eligibility criteria

Inclusion criteria:

1. All participants will require a diagnosis of “mild cognitive impairment due to Alzheimer’s disease” using the most recent NIA and Alzheimer’s Association workshop criteria: (a) presence of memory complaint, (b) Rey Auditory Verbal Learning Test delayed recall (for memory) < 6, (c) Montreal Cognitive Assessment (for global cognition) ranged 18 and 25, and (d) Activities of Daily Living Questionnaire ≤ 30.
2. Intact score for San Diego Brief Assessment of Capacity to Consent (UBACC).
3. If a participant is on memantine, cholinesterase inhibitors, antidepressants, anxiolytics, or vascular risk or disease-related medications (e.g., beta-blocker), the dose should be stable for 3 months prior to recruitment.
4. Age 60–89.
5. English-speaking.
6. Adequate visual and hearing acuity for using mobile-based apps and testing by self-report.
7. Community-dwelling.

Exclusion criteria:

(1) Current enrollment in another cognitive improvement study

(2) Uncontrolled symptoms of major depression

(3) Major cerebrovascular and cardiovascular diseases (e.g., congestive heart failure, pacemaker, prior myocardial infarction)

(4) Neurological diseases (e.g., Parkinson’s disease, multiple sclerosis)

(5) Having an active legal guardian (indicating impaired capacity for decision-making)

(6) MRI contraindication (e.g., pacemaker, claustrophobia)

(7) Color blindness

(8) Alcohol dependency in the past 5 years that is the main contributor to MCI

### Intervention protocol

#### VSOP overview

We will use the INSIGHT online program (Posit Science), including 5 tasks (eye for detail, peripheral challenge, visual sweep, double decision, target tracker) that practice different cognitive processes with processing speed and attention as the shared domain. All tasks are based on visual stimuli and become increasingly more difficult and require faster reaction times as they progress. Participants will respond either by identifying what object they see or where they see it. The training will automatically adjust the task difficulty based on subject’s performance on the past 5 trials (have to reach 75% accuracy rate), ensuring that participants always operate near their optimal capacity. The training programs will automatically record the percentage of completion of each game. The initial training will take longer to allow participants to adapt to the style of cognitive training. Gradually, individuals will practice a shorter length of time to retain the training effect.

#### RFB overview

The RFB protocol will be adapted from the HRVB protocols used in previous studies [28, 29]. The RFB protocol entails a combination of 8 weekly, in-lab training sessions using HRV biofeedback software (Physiocom, Seattle, WA) and daily paced breathing homework using a mobile-based HRV biofeedback app (Inner Balance, HeartMath, LLC, CA). The initial RFB in-lab session (RF identification session; 60 min) introduces the technique of paced breathing. Then, participants are asked to breathe for 2–3-min sets at various frequencies (6 breathes per minute (BPM), 6.5 BPM, 5.5 BPM, 5 BPM, and 4.5 BPM, with a 3-min rest between each paced breathing set) to identify individual RF, the breathing rate at which heart rate is in phase with respiration, that is, increasing/decreasing with inhalation/exhalation, thereby maximizing baroreflex gain and respiratory sinus arrhythmia. The second in-lab session (occurring in the same week) will be 30 min and comprise additional training techniques (abdominal and pursed lip breathing) and fine tuning of RFB rate as needed. Home-based practice (10 min daily) with a mobile app will also be introduced. Subsequent in-lab sessions (occurring weekly across the next 7 weeks) will entail pacer-guided RF breathing and reinforcement of breathing techniques. We propose 10-min sessions of home-based daily practice because Lin et al. successfully adapted an HRVB protocol from Lehrer [28, 30] and Gevirtz [31, 32] implementing a weekly, 6-session HRVB, requesting participants to engage in 10 min of paced breathing at RF once per day. Lin et al.’s protocol instructing 10 min per day of RF breathing resulted in significant effects on HF-HRV outcomes [29]. Of note, standard HRVB training in clinical practice (with cognitively healthy individuals) comprises both visual pacer-guided breathing exercises as well as practice using biofeedback signals (HR and respiration) to try to eventually be able to breathe at RF without a visual pacer (i.e., to “know” and “feel” when one is breathing at RF). For an MCI population, we rely solely on the visual pacer-guided breathing exercises to mitigate the possibility of frustration or inefficacy due to cognitive impairment. As RFB is the proposed driver of peripheral and central effects of HRVB, we propose a focus on this component, both during in-lab training and home-based practice, to ensure translational potential to older adults with MCI.

#### Guided imagery relaxation (IR, control)

Guided IR, equal in dose and frequency to RF practice, will be used to control for relaxation effects that may occur via RFB (which could provide an alternative explanation for outcomes). IR emphasizes using visualization and imagery strategies to help the body relax [33]. A mobile app (Insight Timer, Sydney, New South Wales) will be used to deliver the control intervention.

#### Set-up of the RFB+VSOP intervention

For home-based RFB+VSOP, we will instruct subjects to do 10 min of app-guided paced breathing at RF daily; on select days, there will be VSOP training immediately following RFB. Session duration for the combined intervention will be the sum of paced breathing at RF (10 min) + VSOP (up to 45 min per session). We designed the combined intervention with VSOP immediately following RFB to create the ideal condition for maximizing dose delivery in a format that is convenient for participants.

#### Set-up of the IR+VSOP control group and IR-only group

For the IR+VSOP control group, the control IR strategy will be used, the set-up of which will be the same as the combined intervention group with IR replacing RFB. Participants randomized to the IR-only group will receive weekly in-person check-in visits and perform daily 10-min IR, so that the number of treatment contacts (though not duration) will be equivalent.

#### Weekly in-lab sessions

Orientation and 7 weekly in-lab check-in sessions will be provided in person, and other sessions (including periodical booster sessions) will be self-administered at home. A technical support hotline will be provided. The purpose of these in-lab sessions is to (1) provide orientation of the intervention (week 1 session), (2) ensure the fidelity of intervention across groups (other weekly sessions), (3) maintain motivation across groups, and (4) collect intervention process data. For the RFB+VSOP group, these sessions will begin with a 10-min in-lab RFB training/practice; for the other two groups, 10-min in-lab IR will be provided. For all groups, we will collect resting ECG (5 min) before RF or IR training, and ECG and performance during a 20-min standardized processing speed/attention (PS/A) task (that is format-wise different from VSOP, to reduce the potential training effect of VSOP on the control group). Across-week trajectories within and between groups on these data will be modeled as the training process data.

#### Booster sessions

At 3 and 9 months after completing the initial intervention, a 2-week booster session will be provided. Attending booster sessions has the potential to significantly decrease the incident of dementia further [34].

#### Feasibility of the self-administered intervention

Our completed VSOP efficacy trial and RFB feasibility trial demonstrated a high compliance rate for interventions. We will continue the strategies that help with compliance, including a 24/7 hotline for technical support, check-in sessions during the intervention, and prolonged intervention sessions to ensure subjects’ completion of at least 75% of training. We will also constantly monitor their training activities (VSOP, RFB, and IR control) online.

To assure retention of subjects, we will implement the following strategies. At the program level, we will ensure constant technical support for online VSOP and RFB sessions. The study will reimburse parking costs (and transportation if needed), allow make-up for vacations and illness, and compensate participants and informants for their time. We will make regular follow-up emails or calls during follow-ups. Weekly in-lab sessions will be used for understanding the challenges/barriers participants encounter in their self-administered training sessions. At the staff level, staff will be familiar with the background of each participant, guide participants with sensitivity, ensure frequent communication, address unmet needs, focus on achievement and progress, and establish rapport. Obtaining contact information from surrogates will provide additional routes for reaching out to participants.

#### Criteria for discontinuing or modifying allocated intervention for a given trial participant

The intervention will not be modified for any allocated participants. We may discontinue a participant upon any serious adverse event. Meanwhile, participants can choose to withdraw from the study at any time without affecting their health care, if any, with the University of Rochester.

#### Concomitant care and interventions permitted or prohibited during the trial

We will exclude individuals who are having an experimental pharmacological or non-pharmacological intervention related to cognitive enhancement from enrolling in the study. After enrollment, any participation in such interventions concurrently will be discouraged. If any enrolled participants choose a concurrent experimental intervention, we will record the type of intervention. Any health care as usual will be permitted. The study is regularly recording and updating the type of care and experimental intervention, if any, from the participants, which may be addressed as covariates in later analyses.

### Measures

#### ANS flexibility

ANS flexibility, the primary outcome, will be measured by CAN (central pathway) and HRV (peripheral pathway) at rest and in response to a challenging cognitive stressor, which we term the “stress task.” A protocol comprising rest and a stress task will be conducted within a 3T Prisma MRI scanner with both blood oxygenation level dependent (BOLD) fMRI and MRI compatible ECG monitored simultaneously. All measures for ANS flexibility will be collected at baseline, post-intervention, and 6- and 12-month follow-up.

**Fig. 1:**
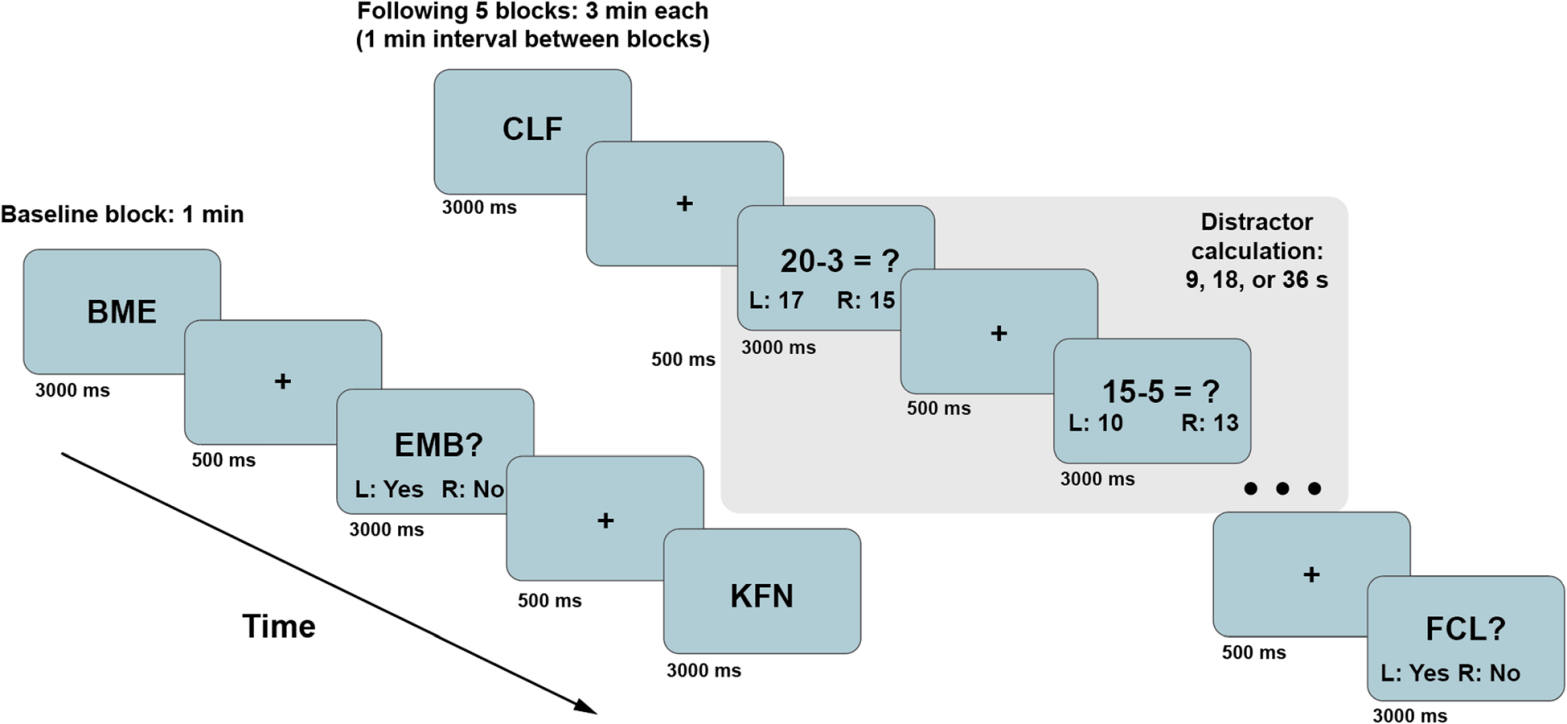
ACT task paradigm. Note. Letter stimuli will be auditorily instead of visually provided

*Auditory Consonant Trigrams* (ACT) [35, 36], a demanding working memory task, will serve as a cognitive stressor (see task paradigm in Fig. 1). Participants receive a three-consonant trigram (e.g., BME) verbally at a rate of one letter per second, followed immediately by a random three-digit number. Participants are asked to recall the trigram after a time delay (9, 18, or 36 s), during which they performed simple arithmetic aloud (counting backwards from the given number by 3’s) to minimize rehearsal effects. The task consists of five randomized trials for each time delay. The length of the task is 15 min. Before ACT, a 10-min acclimation (rest) period will be administered. We selected ACT as the cognitive stress task due to the high cognitive load that can simulate a cognitively demanding circumstance [35].

*Electrocardiography (ECG) data* will be collected during rest and ACT task (both inside MRI) using PrismaFit MRI compatible ECG. Three disposable ECG electrodes will be placed in a lead-II configuration to record the ECG signal. A strain gauge will be secured around the lower chest to record respiration rate, and a pulse oximeter will be applied to the index finger to record pulse rate, which will both be considered as potential covariates in analyses using HRV. ECG signals will be conditioned and collected (1000 Hz sampling; 5 Hz for strain gauge) through an integrated A/D board and written to disk and later scored (with BIOPAC MRI compatible ECG processing software). The signals will be collected continuously during the BOLD imaging sequence. High-frequency heart rate variability (HF-HRV) data will be preprocessed with BIOPAC, using methods described previously [37]. Briefly, consecutive R-R intervals will be preprocessed using a filter at 0.12–0.40Hz for generating HF-HRV, and natural log transformation will be applied. We will process 30–60-s segments [to be consistent with BOLD dynamic functional connectivity (FC) analysis time window size] and remove ectopic beats and artifacts by consistent visual inspection between two raters. The first and last four segments, as well as incomplete segments (i.e., <15 s), of each recording at rest and task will be excluded from the analysis. Null values from motion and arrhythmic artifacts in the remaining data will be excluded by dividing the number of null-absent segments by the total number of segments to obtain the percentage of usable data for each participant. A threshold at 70% will be applied to determine subjects with valid rest and task data.

*Resting-state and task-related BOLD fMRI data acquisition and preprocessing*: Imaging data will be collected using a research-dedicated 3T Siemens Prisma scanner (Erlangen, Germany) with a 64-channel head coil. Each magnetic resonance session begins with a scout image, followed by an MPRAGE scan that provides high-resolution structural-weighted anatomical images for image-registration purposes. A 2D axial fast gradient-recalled echo pulse sequence will be used to generate field maps, to correct for field inhomogeneity distortions in echo-planar imaging sequences. BOLD data will be collected using a gradient echo-planar multi-band imaging sequence. Participants will be instructed to relax with their eyes open without falling asleep. An in-scanner camera will be used to ensure compliance. The first 10 functional volumes from each fMRI protocol will be excluded to allow for signal equilibration effects, then preprocessed using FMRIB Software Library. Images will be corrected for slice-timing acquisition differences, realigned for head motion correction, spatially smoothed by a 5-mm FWHM isotropic Gaussian kernel, and temporally filtered with a band-pass filter (0.01–0.08 Hz). Next anatomical and functional images will be coregistered and spatially normalized to the Montreal Neurologic Institute (MNI) space and resampled into 3 × 3 × 3 mm voxels using trilinear interpolation. *Imaging data analysis—identify SN and SAN*: To identify intrinsic neural networks, we will use resting-state fMRI data across all subjects and all available time points using group independent component analysis (ICA) via the MELODIC algorithm in FSL with a probabilistic ICA approach. An average *z*-score of 3 < *z* < 8 is defined as the threshold for the group ICA maps. SN and SAN will be identified by two raters visually comparing our components to ICA results from other relevant studies [12, 13, 18, 38], for use in the following FC analyses. Furthermore, using the intrinsic networks identified from the resting-state as masks, we will examine the time-dependent change of these networks in response to the ACT task as a measure for ANS in response to the stress task.

*Analysis of weekly in-lab resting and task-related ECG data*: Degree of similarity between candidate ECG features (sliding window based 2-min segments) and previously revealed ECG shapelet will be calculated for each in-lab session. In our previous study, a higher degree of similarity to the ECG shapelet was related to worse learning from VSOP [11]. Here, we will model the trajectory of degree of similarity across in-lab sessions and calculate the relationship between the slope of trajectory and intervention effects on outcomes. If the combined intervention has greater, additive effects, compared to other groups, on outcomes, we expect to see a stronger relationship between the training process data and changes of main outcomes in the combined intervention group.

Cognition (primary outcome), NPS, IADL, and neurodegeneration (secondary outcomes) will be collected at baseline, post-intervention, and 6- and 12-month follow-up. Blood biomarkers (other outcomes) will be collected at baseline, post-intervention, and 12-month follow-up. All these measures together are considered as the markers of the progress of dementia.

*Cognition*: Executive function and episodic memory are two primary domains affected earliest by AD-associated neurodegeneration [39]. Executive function will be measured using EXAMINER, a computerized test package designed for RCTs. It includes 8 tests and calculates 4 sub-domain composite scores on working memory (Dot counting and N-back), inhibition (Flanker, Continuous Performance Test, and Anti-saccades), cognitive control (Dimensional Set Shifting), and Fluency (Phonemic Fluency and Category Fluency), and an overall composite score for executive function [40]. Test-retest reliabilities are .78 to .93 [41]. EXAMINER has 3 alternative packages to reduce practice effects and embed basic cognitive process domains (e.g., PS/A) in every task. We do not plan to separately measure these domains using Useful Field of View (UFOV), because UFOV is very similar to VSOP and improves significantly with VSOP [42–44]. Episodic memory will be assessed using the Rey Auditory Verbal Learning [45] and Brief Visuospatial Memory tests [46]. These tests are similar in format, but one focuses on object naming while the other on object shapes, allowing a comprehensive assessment of visual and verbal memory. We chose these standard and validated clinical tests to ensure that our results are comparable to the literature and relevant to practice. We will use alternative forms of the tests to reduce practice effects [47]. The *Z*-transform scores across all assessment points within each test will be developed first to derive 2 composite scores (learning and delayed recall). Both assessments have been validated in MCI and differentiate individuals with MCI from healthy counterparts [45, 46]. A composite score synthesizing EXAMINER composite score, learning, and delayed recall will be created and used as the primary measure for cognition.

*NPS* will primarily be measured using the informant-rated neuropsychiatric symptom inventory (NPI), the full version of 12 domains [48], including both frequency and severity (based on present symptom) scores in the past month.

*IADL*: Timed IADL objectively measures performance speed and accuracy on multiple IADL domains [24, 49]. Time spent on each task will be recorded with adjustment on whether an individual accurately completed each task. Average completion time across the tasks will be used as the outcome measure. To reduce practice effects, alterative items within each category (e.g., pill bottles for different conditions with different instruction labels) will be used.

*AD signature cortical thickness*: Structural MRI will be collected along with BOLD fMRI described in the ANS flexibility measure. Each session begins with a scout image, followed by an MPRAGE scan with 1-mm isotropic resolution to provide high-resolution structural-weighted anatomical images. 3D FLAIR and susceptibility-weighted images (SWI) will be acquired to ascertain brain pathology (e.g., cerebrovascular disease). Our structural MRI measure is a composite of AD signature cortical thicknesses segmented and analyzed with FreeSurfer, which is composed of the following individual regions of interest: precuneus, fusiform, and inferior and middle temporal lobes (all contributing to AD signature cortical thickness).

*AD conversion and reversion to normal*: AD conversion is defined as meeting clinical or MRI-pathological criteria for AD [50]. If pathological conversion is observed (e.g., degree of atrophy worsens), a consensus clinical diagnosis of AD will be determined by the PIs and Co-Is using the 2011 probable AD diagnostic criteria [51]: memory deficit (≥ 1.5 SD below age- and education-corrected norms), MoCA < 20, decline in activities of daily living, and cognitive changes not due to other conditions. If a clinical conversion is determined, we will emphasize it was for research purposes and refer the participants to their providers. *MCI reversion to normal cognition* will be assessed and treated similarly as AD conversion. At the 12-month follow-up, a final classification will be determined for each participant (e.g., constant as MCI, conversion to AD, reversion to normal) based on the AD Neuroimaging Initiative criteria [52].

*Blood collection*: We will implement the following rules in blood collection in order to reduce pre-analytical variations that could affect biomarker levels: (i) collect blood samples after at least 8 h of fasting (only water and medications are allowed), at the same time in the morning (between 8:30 am and 10 am) [53], and after the participant has been sitting for at least 10 min; (ii) obtain information on medications, infection, vascular disease conditions [54], and unsupervised leisure activities outside the trial, and record the time between last consumption of any food or drink and time of blood collection, as well as blood collection time [55]; and (iii) collect blood at least 24 h after the last intervention session [56, 57] at post-intervention, in order to mitigate any effects on biomarkers from the last bout of intervention. On the day of blood collection, trained phlebotomists will collect blood following a venous blood collection protocol. If a participant has forgotten to fast, the phlebotomist will notify the project managers, who will attempt to reschedule the blood draw. The phlebotomist will collect a total of 20 mL of blood, half into a 10-mL plasma (EDTA-treated) tube and the other half into a 10-mL serum tube. A lab technician will process and aliquot these specimens according to an established protocol. Briefly, the plasma and serum tubes will be gently mixed and centrifuged at 4°C using a temperature-controlled centrifuge (i.e., Sorvall Heraeus Multifuge 3 S-R) with a Swing out Rotor (i.e., #75 006 445) at 1439 g for 15 min. The tubes will be removed from the centrifuge immediately after completion. From the plasma tube, up to eight plasma aliquots of 500 μL each will be made; from the serum tube, up to six serum aliquots of 500 μL each will be made; packed cells from each plasma tube will be transferred into a 2-mL aliquot. The aliquoted samples (i.e., plasma, serum, and packed cells separated from the plasma) will be stored in a −80°C freezer. *Biochemical analyses*: Simoa is an ultra-sensitive method measuring blood protein biomarkers [58], which can be measured using Quanterix SR-X analyzer at high precision [54]. Simoa assays have been recently used in epidemiological studies to measure blood neuropathological biomarkers [54, 59–62]. In this study, we will use commercially available Simoa assays to measure t-tau, p-tau, neurofilament light, Aβ40, and Aβ42 in plasma samples. These are emerging plasma-based beta-amyloid markers with high accuracy [63].

*Covariates* will be assessed by collecting information on participants’ background characteristics (i.e., demographics), current medications, and subjects’ engagement in leisure activities and use of stress relief/relaxation-related activities outside of the study.

### Study timeline

Study preparation will take 3 months, enrollment will take 3 years, and data collection will be completed within 4.5 years.

### Sample size

Sample estimation is based on the effect of RFB+VSOP combined intervention on two primary outcomes (ANS flexibility and cognition/IADL) by comparing to other groups (IR+VSOP control and IR control only). Literature indicates the following information related to deciding the effect size: (1) a moderate to large effect (using Hedges’ *g* = 0.83) of HRVB (including RFB) on stress regulation measures [17], (2) a small to moderate effect of VSOP on cognition, (3) correlation between ANS measures (*χ*^2^ = 11.70, *r* = 0.44) [7, 64], and (4) correlations between ANS measures and cognition (pooled *r* = 0.16) [65]. We estimate alpha = 0.025 (correct for two comparisons: combined intervention group with VSOP+IR control, as well as with the IR control group), and 3 measurement time points: baseline, post-intervention assessment, and 12-month follow-up. A sample size of 96 will be able to detect a small effect (*d* = 0.20). We further estimate a 20% attrition rate at the 12-month follow-up (in another local longitudinal study of cognitively healthy older adults Dr. Lin conducted [66], the attrition rate was less than 20% for up to a 5-year follow-up), and we will use a sample size of 114 (*n* = 38 per group).

### Recruitment

Various strategies of clinical and community recruitment will be conducted. We have been and will continue utilizing our research participant registry, AD-CARE research program, Memory Care Program, Older Adult Outpatient Clinic, and Greater Rochester Practice-Based Research Network. Together, we have access to over 3000 potentially eligible MCI patients. So far, we have successfully recruited over 500 older adults for multiple NIH observational or intervention studies.

### Allocation

Age (60–75 vs. 76–89) will be the factor for stratification. A block-based randomization strategy will be utilized. Co-I (Z.Z.) will create a randomization schedule in REDCap using a random-number generator. Qualified participants will be randomized after completing baseline data collection. The study staff who is not conducting post-intervention assessments will conduct the randomization procedure.

### Blinding

We have built in 4 strategies to ensure blinding: randomization using permuted blocks, all investigators blinded to group assignments (except the biostatistician Z.Z.), all participants blinded to the study aims and reminded as needed not to discuss their experiences with the outcome assessor, and outcome assessors blinded to the study aims, group assignments, and previous test results, and to interact with participants only for data collection [67]. Assessors will not interact with other staff who are not blinded and will participate in separate meetings. Blinding success will be assessed after each data collection: Was group assignment unveiled? Why? For participants whose group assignments are revealed, a different blinded assessor will collect subsequent data. Other strategies include the use of standardized intervention protocol, forms, and reliable and valid variable measures. The analyses will incorporate all design features (e.g., repeated measures within persons, missing data). Unblinding to investigators and study team will only occur when the data analysis of the primary outcomes is completed.

### Data collection method

Data collectors will have checklists to follow for the data collection visit protocol (e.g., consent obtained, date, participant code, etc.). This checklist will be then reviewed by the Project Coordinator who will monitor for deviations from the protocol. The Project Coordinator will check the first 3 enrollment and consent visit checklists of each data collector. Thereafter, he/she will randomly select 15% of the checklists every data collection cycle (6 months). The project coordinator will generate reports for the PIs to review every data collection cycle. Data collected will be kept in a master data set maintained by the project coordinator. Queries and reports will be run periodically over the course of the study related to the integrity of the data, outliers, missing data, etc. The project coordinator will generate a report after each data collection cycle (every 6 months) that will be discussed with the PIs and project statistician. Any necessary systems change will be evaluated at each of these meetings.

### Data management method

Names and other personal identifying information will be confidential. Rigorous procedures will be in place to safeguard and eventually destroy identifiable information to protect the identity of participants. We will keep data collected from participants, written informed consents, and participant contact information in locked filing cabinets in restricted-access office spaces. Data from questionnaires will be identified only by study ID. Electronic datasets will be stored only on a password-protected server within the University. Databases will be housed on secure UR servers. The servers are in a physically secure location and are backed up nightly, with the backups stored in accordance with the retention schedule of daily, weekly, and monthly tapes retained for 1 month, 3 months, and 6 months, respectively. Weekly backup tapes are stored offsite. Computers will be safeguarded from theft and damage (e.g., locks on physical access, virus protection and encryption programs), and no data will be stored in a portable computer. Project files and databases associated with the study will only be available to research personnel through the authorization of the PIs. In addition, study reports (such as aggregated data in progress reports) generated by the research team will provide total anonymity because no names or identifying information will be part of such reports. Participants and staff will be apprised of their rights and responsibilities under the Privacy Act of 1974, including penalties for violations. All staff involved with the research project will receive training on their function, roles, and responsibilities to protect and maintain the privacy and confidentiality of research participants, and will complete NIH-approved training in this area.

Any amendment versions of IRB documents will be kept. Blinding related to randomization is kept in a password-protected file in our server with selected unblinded staff having access. Project files and databases associated with the study will only be available to research personnel through the authorization of the PIs. The UR servers are backed up nightly, with the backups stored in accordance with the retention schedule of daily, weekly, and monthly tapes retained for 1 month, 3 months, and 6 months, respectively. Weekly backup tapes are stored offsite.

Measures for use in the study were selected carefully based on their established psychometric properties for questionnaires, and standardized protocols will be used for measuring brain imaging, blood, and ECG data. All measures will be administered by study staff, Center for Advanced Brain Imaging and Neurophysiology (brain imaging) and Clinical Research Center (CRC, blood) staff, respectively. Data collection procedures will be standardized based on a detailed protocol, and data collectors will be trained to ensure consistency in the procedure. Twice a year, the team will review procedures with all the staff involved in data collection to prevent deviations from the protocols. Finally, all data will be entered into REDCap or a statistical database. The data collection process will entail site staff entering data both directly into databases, using source worksheets (e.g., chart abstract data entered onto data collection source worksheets), and into REDCap directly. Data will be stored in a password-protected management information system and in SPSS databases, which are maintained on a secure UR server. Once data have been verified against source documents, any data entry errors are corrected and data are moved to an archived file. Data entered are immediately stored in a study database where they are accessible for review by the study team. Our data collection project will rely on a thorough study-specific data dictionary defined in an iterative self-documenting process by all members of the research team. Blood samples will be processed as per protocols in the CRC for storage in preparation for analysis.

Staff members will not be allowed to abstract data, interview a subject, or process data from a subject that they know personally. Identification codes rather than names will be used on the data collection forms. The PIs will assure that survey practices adhere to the provisions of the US privacy act of 1974 with regard to surveys of individuals for the Federal government. The handling of data will be limited to numerical values and statistical summaries. Identifiers linking identification codes with individual names are only available to the PI and study staff who contact participants. Data collection into REDCap is stored on a protected server. Personal computers are logged off at the end of each work day to prevent inadvertent access to the network and data stored on the computer. Data will be stored on a protected server and the access will be limited by a password-protected network subdirectory where sensitive data are stored. Sensitive data will not be sent via email. Blood samples identified by code will be stored in a −80°C freezer in the SON level 2 safety biomolecular lab that has limited access.

### Data monitoring

At a minimum, an independent safety monitor (ISM) will conduct reviews early in year 1 and each year of the project thereafter. The first review will be conducted before the formal start of enrollment, reviewing documents (i.e., protocol and data safety monitoring plan) related to the study. In each of the following meetings, ISM will randomly audit 10% of enrolled cases during the period (i.e., between last and current review meeting); the auditing will include reviewing all assessment, intervention, and data management documents to determine if the procedure is aligned with the IRB protocol. Additional meetings with the study team or additional information from the study team will be provided on the recommendation of the ISM. After each meeting, a summary of recommendations made by the ISM as appropriate and (if applicable) the action plan for response will be submitted to NINR.

The study investigators will submit statistical reports to the ISM 1 week prior to the scheduled review. These reports will include all reported data up to and including 14 days prior to the reporting deadline (except for serious adverse events, which are to be reported within 24 h of an event). For each review at which the study is to be considered or monitored, the PI will present an overall progress statement. This brief statement will contain any IRB-approved protocol modifications, the assurance that the study investigators have considered the study’s progress and that there is/is not evidence of safety issues that should be addressed by the ISM. Data will be presented in a blinded manner during the open sessions of the ISM meetings. Data and discussion are confidential. Participant identities will not be known to the ISM. Yeates Conwell, MD, Director of the Aging Research Office, University of Rochester, is the ISM, approved by the funder.

Of note, the interim analyses and decision to terminate the trial will only be initiated and justified by ISM and approved by the funder.

### Ethics and dissemination

The protocol has obtained approval from the University of Rochester Research Subject Review Board.

#### Verbal consent for phone and in-person screening

When potential participants contact us, the study staff will first explain the study and obtain their verbal consent to proceed with phone screening, using the phone screening script. Compliance with HIPIAA authorization at the time of screening is being obtained through alternate HIPIAA language. A waiver of documentation of consent has been requested for this step because it is conducted over-the-phone so participants will not be able to sign this consent themselves.

#### Initial signed paper consent and re-consent

The initial signed paper consent will be obtained during the in-person interview by the subject.

1. Explanation of the study: During the in-person interview, the staff will explain the study in detail to the participants, including the study purpose, procedures, time commitment, randomization, data collection, risks, benefits, privacy, confidentiality, compensation, voluntary nature, and contact persons for questions and concerns. Emphasis will be placed on explaining the risks. Questions will be answered and any confusion about the study will be resolved.
2. Assessment of capacity to consent: The staff will assess the potential intervention subject’s capacity to consent using the UCSD Brief Assessment of Capacity to Consent form (UBACC) for the study. A score of 2 on items 1, 2, 4, 6, 7, and 9 is needed for inclusion in the study. If a participant score is less than 2 on any item, the staff will re-explain the study and reevaluate the capacity of the subject. Given the potential fluctuation of their cognitive capacity, if a subject still fails to score 2 on required items, we will ask the person to return on another day to re-take the UBACC. If the subject still scores below 2 on any required item during the 2nd visit, he/she cannot be enrolled in the study. Of note, other items than the required 6 items are provided for education purpose. If they do not answer 2 on those items, the staff will re-explain the study, but such failures will not interfere with their eligibility in the study.

##### Ancillary and post-trial care

If the study team concerns about any participants’ mental health or safety, the study team may contact their primary care physician or other relevant health care provider. The study team will notify participants of these concerns prior to contacting their providers.

### Data analysis

#### Determine the intervention effect on outcomes

All primary analyses will follow *the intention-to-treat principle* (e.g., group assignment in the analysis will be based on randomized group assignment regardless of the level of adherence to provide unbiased comparisons of the effects among the groups). This principle will account for any potential data loss. We will fit linear mixed-effect models for each outcome measure, accounting for repeated measures at baseline, post-intervention, and 6- and 12-month follow-up. The models will include fixed effects for data collection visit (categorical variable), group assignment (compare the combined group with other groups), visit and group interaction, respective baseline outcome measure, and covariates identified as being imbalanced among groups. Stepwise variable selection using Akaike Information Criteria will be used to select the fixed effects for inclusion. A random participant-specific effect will be included to account for correlation between visits in the same participant. The model-based average within-group change in outcome measure for each group will be computed at each post-intervention and follow-up point. This will allow us to rank the groups in terms of average within-person gains in each outcome measure. In addition, *the effect sizes of RFB+VSOP and IR+VSOP control for each outcome measure* will also be calculated using standardized mean difference with 95% CI [(*M*_int_ − *M*_control_ at later time) − (*M*_int_ − *M*_control_ at baseline)]/intra-participant standard deviation; “int” refers to combined intervention or VSOP; “control” here refers to attention control. The effect size of RFB+VSOP will be compared with that of the IR+VSOP control group to determine the additive effect.

#### Determine the relationships between changes of outcomes

We will first examine correlations between the changes of central and peripheral measures of ANS at rest and reactivity, respectively, from baseline to later time points for the entire sample. Next, we will use the mixed-effect model to determine the main and interaction effects of change in ANS measures (primarily, reactivity) and group on changes of each dementia progress measure (comparing baseline to later time points). Performing this analysis is to determine whether a change in ANS flexibility will lead to greater improvement in dementia progress measures in the combined intervention group, compared to other groups.

## Discussion

We capitalize on adding RFB practice to VSOP to modify ANS flexibility, which we in turn hypothesize will further strengthen the effect of VSOP on multiple markers of dementia progress. Adaptation capacity to environmental demands contributes to long-term cognitive and functional maintenance in old age regardless of the effect of neurodegeneration. Therefore, enhancing ANS flexibility, essential for adaptation capacity, may impact the long-term cognitive and functional outcomes in old age, including those at risk for dementia. RFB provides a bottom-up modulation that may directly enhance the neurobiological adaptation during VSOP training that is required for neuroplasticity. Further, along with enhanced neuroplasticity and cognitive outcomes, we suggest that combining RFB and VSOP will strengthen overall adaptation capacity better than VSOP alone. Together, these effects are hypothesized to slow dementia progress. Such a combined intervention approach is novel, but grounded in a solid mechanistic understanding.

Our work is inspired by a novel conceptual framework of “physiological geroscience,” which proposes a primary-prevention approach to addressing aging-associated physiological factors that in turn can slow aging and extend the healthspan [68]. Seals et al. suggest that optimizing physiological function via lifestyle interventions, pharmaceuticals, etc., in the aging process can slow down the decline of other interrelated functions (e.g., cognitive function and neurodegeneration) and delay the incidence of aging-associated diseases (e.g., dementia). We are among the first to apply this approach to examine whether modifying ANS flexibility, and therefore increasing adaptation capacity, can slow the progress of dementia. The components of ANS flexibility are vulnerable to aging. Vascular-wise, aging-related decline in endothelial function leads to changes in baroreflexes [69]; neurologically, aging-related decline is implicated in the decreased neural efficiency of central autonomic networks [70]. Both mechanisms can cause the disruption of the ANS flexibility [10, 71], which in turn affects the regulation of self-oriented behaviors, including stress adaptation.

Furthermore, the therapeutic implication may be different between studying primary- (e.g., adaptation capacity) vs. secondary- (e.g., hippocampal atrophy) prevention factors for the progress of dementia. This is crucial for aging populations since they are often exposed to heterogeneous health conditions that impose secondary adverse effects on the progress of dementia. Enhancing common preventative factors may strengthen cognitive and brain function across different conditions in which secondary-prevention factors can be too heterogenous to be modified via a common pathway.

Adaptive cognitive training programs hold great promise for slowing progression to dementia in older adults with MCI. The research proposed here translates the growing understanding of the top-down and bottom-up regulation of integrated central and peripheral systems to bolstering the transferred and long-term effects of cognitive training and reducing the burden of dementia for patients, caregivers, and the health care system. Additionally, findings from the proposed study may have implications in addressing the long-lasting public health concerns on the impact of maladaptation to stress on neurodegeneration [72]. After completion of the clinical trial, relevant results will be presented in community settings and published in peer-review research journals. Further stages of clinical trials (e.g., stage 3) will be proposed, or modified if needed, to test the effectiveness of the intervention.

## Data Availability

We will create a database for hosting all data at the University of Rochester (UR) and managed and stored by the UR Information Analyst on-site. A final complete database will be created to host all data which will be stripped of any identifiers and stored pursuant to UR Institutional Review Board protocols. After data entry, cleaning, and linkage, the Information Analyst will provide the research team a de-identified data set for purposes of analysis and manuscript preparation. We will make the data and associated documentation available to users only under a data-sharing agreement as suggested by the NIH that provides for (1) a commitment to using the data only for research purposes and not to identify any individual participant, (2) a commitment to securing the data using appropriate computer technology, and (3) a commitment to destroying or returning the data after analyses are completed. The richness of the data, combined with the interdisciplinary nature of the research team, provides opportunities for future wide disseminations. The interdisciplinary team has a strong history of presenting at local, state, and national conferences that span the fields of neuroscience, gerontology, cognition, and psychophysiology. It is of the utmost importance to this team that the work products from this study will be disseminated in a manner that provides implementation in the field.
